# Transomics2cytoscape: an automated software for interpretable 2.5-dimensional visualization of trans-omic networks

**DOI:** 10.1038/s41540-024-00342-8

**Published:** 2024-02-19

**Authors:** Kozo Nishida, Junichi Maruyama, Kazunari Kaizu, Koichi Takahashi, Katsuyuki Yugi

**Affiliations:** 1 Laboratory for Integrated Cellular Systems, RIKEN Center for Integrative Medical Sciences, 1-7-22 Suehiro-cho, Tsurumi-ku, Yokohama, Kanagawa 230-0045 Japan; 2 Department of Biotechnology and Life Science, Tokyo University of Agriculture and Technology, 2-24-16 Nakamachi, Koganei-shi, Tokyo 184-8588 Japan; 3 Center for Biosystems Dynamics Research (BDR), RIKEN, 6-2-3 Furuedai, Suita, Osaka 565-0874 Japan; 4 Institute for Advanced Biosciences, Keio University, Fujisawa, 252-8520 Japan; 5 Department of Biological Sciences, Graduate School of Science, The University of Tokyo, 7-3-1 Hongo, Bunkyo-ku, Tokyo 113-0033 Japan

**Keywords:** Biochemical networks, Software

## Abstract

Biochemical network visualization is one of the essential technologies for mechanistic interpretation of omics data. In particular, recent advances in multi-omics measurement and analysis require the development of visualization methods that encompass multiple omics data. Visualization in 2.5 dimension (2.5D visualization), which is an isometric view of stacked X-Y planes, is a convenient way to interpret multi-omics/trans-omics data in the context of the conventional layouts of biochemical networks drawn on each of the stacked omics layers. However, 2.5D visualization of trans-omics networks is a state-of-the-art method that primarily relies on time-consuming human efforts involving manual drawing. Here, we present an R Bioconductor package ‘transomics2cytoscape’ for automated visualization of 2.5D trans-omics networks. We confirmed that transomics2cytoscape could be used for rapid visualization of trans-omics networks presented in published papers within a few minutes. Transomics2cytoscape allows for frequent update/redrawing of trans-omics networks in line with the progress in multi-omics/trans-omics data analysis, thereby enabling network-based interpretation of multi-omics data at each research step. The transomics2cytoscape source code is available at https://github.com/ecell/transomics2cytoscape.

## Introduction

Visualization of large-scale molecular networks and comprehensive data is an essential technology to facilitate the interpretation of ‘omic’ data^1^. To date, a variety of network visualization software has been developed, such as VANTED^2^ for metabolomic data in the context of metabolic pathways and Cytoscape^3^ for protein-protein interactions and other types of networks.

Yugi and colleagues have established a ‘trans-omics’ analysis method that allows the reconstruction and visualization of biochemical networks spanning multiple omic layers^4^. Their principle for visualizing the trans-omic network is to provide interpretability with the support of researchers’ prior knowledge. The prior knowledge used to visualize interpretable networks has two components: (1) conventional pathway map layout and (2) stacked omic layers with a 2.5D perspective, which is an isometric view of the stacked layers in a 3D space. A significant advantage of 2.5D visualization is that it enables interpretable visualization of multiple pathways that belong to different omic layers altogether, with regulatory edges connecting the multiple pathways. This framework allows for conserving the conventional layouts of the multiple pathways that consequently provide interpretable visualization of the multi-layered regulatory networks. A disadvantage of 2.5D visualization is that some of the nodes and edges are hidden under the layer planes. Also, the visualization of trans-omic networks was dependent on personal and time-consuming efforts, especially in the case of preparing a submission-ready quality figure. Accordingly, it was not realistic to visualize trans-omic networks at each step of a multi-omics/trans-omics research project, which would allow one to refer to a preliminary image of the network for the further characterization of molecular mechanisms in subsequent steps.

Here, we present an R package, transomics2cytoscape, that automates the visualization of trans-omic networks from tabular data describing network connectivity. We specified four requirements for the human-interpretable and reproducible visualization of multi-layered biochemical networks, i.e., trans-omics networks: (1) importing pathway maps from databases, (2) stacking pathways as layers, (3) drawing edges between stacked layers, and (4) automation of workflow. The transomics2cytoscape package satisfies these four requirements, and reproducibly generates images of the trans-omic networks in the context of conventional pathway map layouts and stacked omic layers. In line with recent developments in workflow automation, transomics2cytoscape allows one to bypass human-dependent processes in the visualization of trans-omic networks. In this article, we present an overview of transomics2cytoscape along with two use cases visualizing previously published trans-omic networks. Finally, we conduct an extensive evaluation, covering a wide range of existing tools, based on the requirements for the trans-omic network visualization, to strengthen the effectiveness of transomics2cytoscape.

## Results

### Software overview

**Fig. 1:**
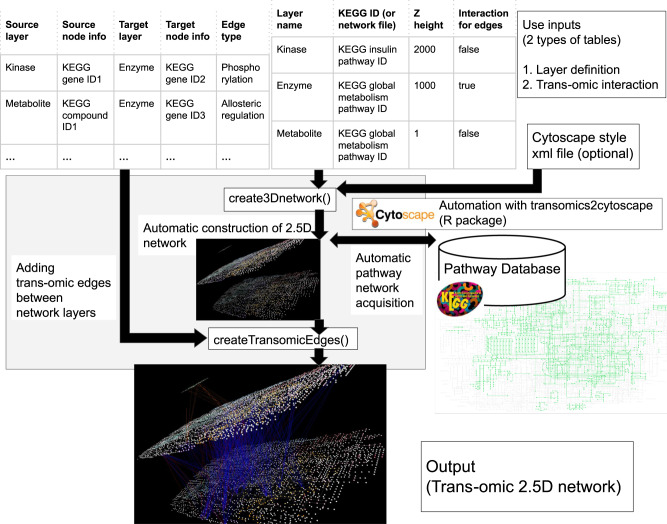
The transomics2cytoscape visualization workflow. The user is expected to prepare table files in two formats. One is an input for the “create3Dnetwork” function to create multiple omic layers, and the other is an input for the “createTransomicEdges” function to create edges for the trans-omic interactions drawn by create3Dnetwork. The “create3Dnetwork” function can automatically import network layouts from the KEGG database by entering the pathway ID information in the table. The user can change the default style by preparing an XML file for a different Cytoscape style.

We developed the R package transomics2cytoscape that automates the 2.5D visualization^5,6^ workflow for trans-omics studies. transomics2cytoscape has two types of input table files and two functions that process and visualize each of the input table files. An understanding of these file formats and how to prepare the data is required to use transomics2cytoscape. One of the two input table files is called the “layer definition table file”, which specifies the pathway network of each layer and its Z-coordinate, and the other file is called the “trans-omic interaction table file”, which defines the interaction that connects each layer (Fig. 1, top). Hereafter, we refer to an interaction between omic layers as a “trans-omic interaction”. Given the layer definition file as an input, create3Dnetwork draws multiple layers in 3D space.

**Fig. 2:**
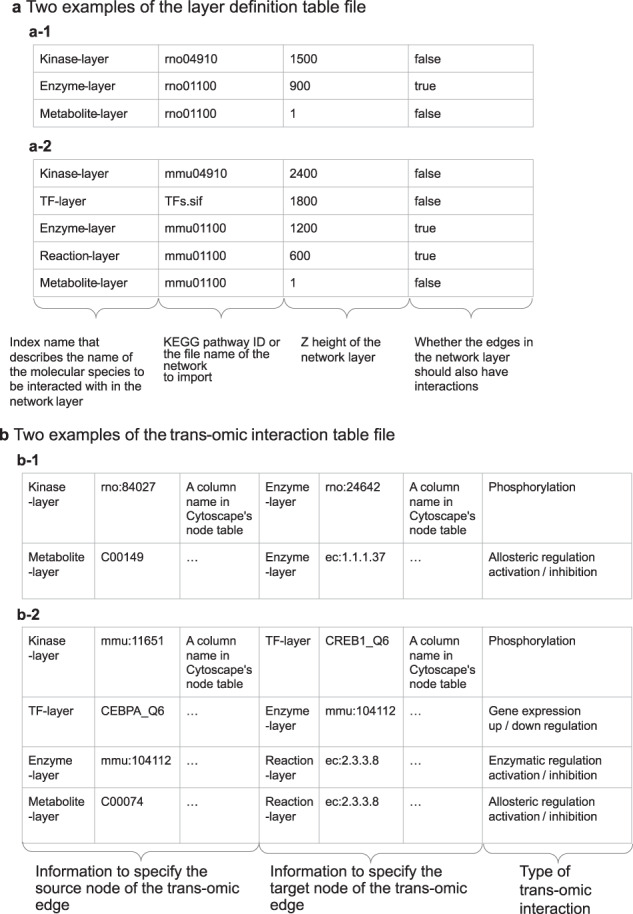
Description of input table formats of transomics2cytoscape. **a** Two examples of the layer definition file. The first column is for an index for each layer. The second column is for a KEGG pathway ID or a filename that Cytoscape can import. The third column is the position of each network layer in the Z-coordinate. The fourth column indicates whether a node should be created at the center of an edge. In this example, we provide molecule types as the layer index. **b** Two examples of a trans-omic interaction table file. In the first three columns, transomics2cytoscape requires information specifying the source nodes of the trans-omic interaction. The next three columns are used for information specifying the target nodes of the trans-omic interaction. The seventh column describes the type of trans-omic interaction.

The data format of the layer definition table file is a four-column tab-delimited text file (Fig. 2a). The first column is for an arbitrary character string representing an index for each layer. This information is also used in the trans-omic interaction table file. The second column is expected to contain a KEGG^7^ pathway ID or a filename in the file format that Cytoscape can import. For KEGG, there is no need to prepare file data; just write the KEGG pathway ID, and transomics2cytoscape will retrieve the pathway data from KEGG. If a user needs to import original network data other than KEGG, the user should place a Cytoscape-compatible file in the networkDataDir path, which is one of the arguments of the create3Dnetwork function. The third column is the position in the Z-coordinate of each network layer. The fourth column is a Boolean value indicating whether or not trans-omic interactions should be provided with the edges as well as the nodes. Trans-omic interactions apply not only to edges from a molecule (node) in a layer to a molecule (node), but also to edges from a molecule (node) in a layer to an interaction (edge) in a layer. For example, the global metabolic pathway map of KEGG (https://www.genome.jp/pathway/map01100) does not have nodes to represent molecular interactions (i.e., metabolic reactions). By setting the fourth column to “true”, transomics2cytoscape adds a node at the midpoint of a network edge in the layer. By changing the network layout, the user can also visualize the node-to-edge (and the opposite) and edge-to-edge interactions, which cannot be done with the original KEGG network layouts. The number of layers in this table file can be increased to any number. Fig. 2a shows examples of trans-omic networks with three layers (a-1), and five layers (a-2).

The trans-omic interaction table is a tab-delimited text file with seven or more columns (Fig. 2b). The first to third columns and the fourth to sixth columns contain information about the source (start) and the target (goal) of the trans-omic interaction, respectively. The first and fourth columns are character strings corresponding to the first column of the layer definition file: the source layer and the target layer of the trans-omic interaction, respectively. The second column is for designating the ID of the source node of the trans-omics interaction. The third column specifies the column name in the node table of Cytoscape. The fifth and sixth columns are the same as the second and third columns, except that the fifth and sixth columns are for the target node of the trans-omics interaction. The seventh column is for an arbitrary character string that describes the attribute value of the interaction between layers (e.g., “metabolite_activator” or “metabolite_inhibitor”). Figure 2b-1 and b-2 are the trans-omic interaction table files for the layer definition table files in Fig. a-1 and a-2, respectively.

**Fig. 3:**
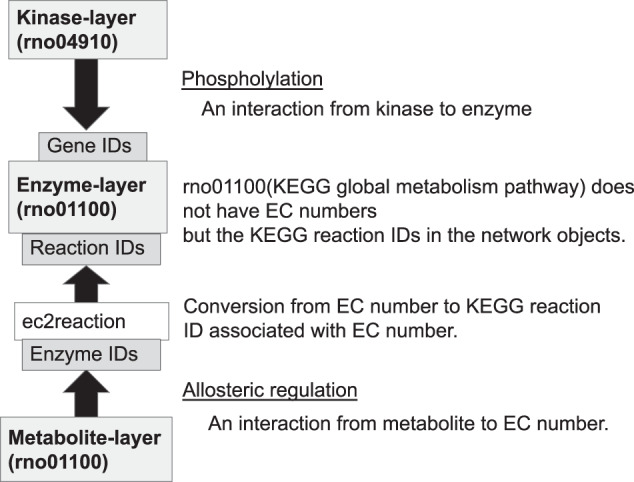
Bridging multiple ID namespaces for an identical enzyme targeted by different types of trans-omic interactions. The ec2reaction function converts an EC number into a KEGG reaction ID to integrate trans-omic interactions that have different IDs for the same enzyme. For example, the target ID for regulatory metabolite-protein interactions available in BRENDA is an EC number, whereas the target ID for phosphorylation of metabolic enzymes is typically a gene ID. Conversion of IDs, such as from an EC number to a KEGG reaction ID, allows connecting these trans-omic interactions to the metabolic enzymes that they target.

Molecular species in the same layer may belong to different ID namespaces depending on the contexts in which the entities are specified, for example, as an enzyme protein or an enzymatic reaction. The namespace of the target IDs for metabolite-protein interactions, which spans from the metabolite layer to the enzyme layer, is the EC number (Fig. 2b-1), because the BRENDA^8^ database, a major data source for metabolite-protein interactions, specifies each enzyme reaction by an EC number. The namespace of the target IDs of protein phosphorylation, which spans from the kinase layer to the enzyme layer, is the ID of the enzyme protein, which is typically provided after proteomic measurements^9^ (e.g., UniProt^10^). Also, the KEGG global metabolic pathway map, which transomics2cytoscape expects to be one of the most frequently used data sources for a network layer, does not use EC numbers but KEGG reaction IDs for metabolic enzymes. transomics2cytoscape converts EC numbers to KEGG reaction IDs using the ec2reaction function, so that it can connect trans-omic interactions of different modalities that meet at the metabolic enzyme layer (Fig. 3).

### Case Study I: Insulin action across the phosphoproteome and metabolome in rat hepatoma Fao cells

**Fig. 4:**
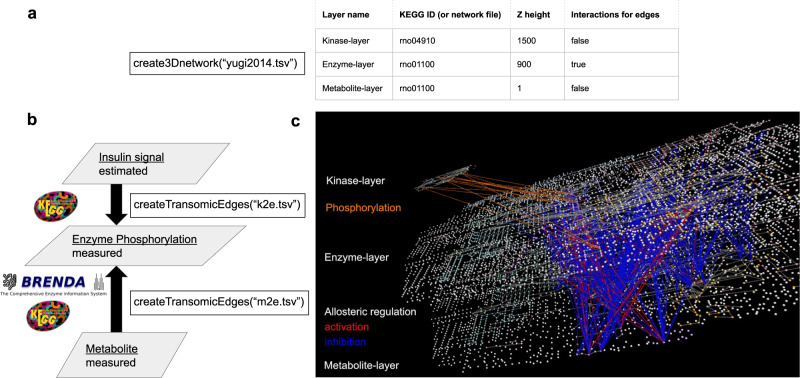
A three-layered trans-omic network of insulin action in rat hepatoma Fao cells automatically generated by transomics2cytoscape. **a** A layer definition table file (‘yugi2014.tsv’) that defines names, positions (‘Z height’), etc. of the three layers. The metabolites in the global metabolic pathways from KEGG (rno01100) are positioned between X-coordinates 0 and ~4800. In the network figure (**c**), the Z-coordinate of the top layer is set to be 1500, which is approximately one-third of the length of the X-coordinate of rno01100. The Z-coordinate of the middle layer was set to be 900, slightly higher than the midpoint between the bottom and top layers. **b** The arguments to the createTransomicEdges function, such as ‘k2e.tsv’ and ‘m2e.tsv’, are names for trans-omic interaction table files expected to contain kinase-substrate relationships and metabolite-protein interactions, respectively. **c** The trans-omic network of insulin action in rat hepatoma Fao cells^6^ automatically produced by transomics2cytoscape. The top layer is the insulin signaling pathway imported from KEGG (rno04910). The middle and bottom layers are the global metabolic pathways from KEGG (rno01100) for enzymes and metabolites, respectively. The nodes in the network are defined in the same way as in KEGG. Spheres represent metabolites and other molecules and cubes represent gene products, typically proteins. Additional nodes representing enzymes were drawn at the midpoint of all reaction edges of the global metabolic pathway map (rno01100) since it does not contain any nodes for enzymes, but only edges. As for the edge color between layers, orange indicates protein phosphorylation. Red and blue indicate activation and inhibition of an enzyme by metabolite-protein interaction, respectively.

First, we show an example of visualizing a trans-omic network for acute insulin action in rat hepatoma Fao cells^4^. This network does not include gene expression layers but includes metabolic regulatory pathways that traverse the phosphoproteome and metabolome layers. transomics2cytoscape provides an automated workflow that facilitates creating a 2.5D network from the table data. We created the trans-omic network of insulin action (Fig. 4) using datasets in the folder named ‘usecase1’ at 10.5281/zenodo.8201898 and codes in the folder named vignettes at 10.5281/zenodo.8201898. To reproduce the figure, the following operations must be performed after the transomics2cytosape code is complete. 1. Zoom out by turning the mouse scroll wheel down until all network nodes become visible. 2. The network does not appear in a layered view only by zooming out because the user is looking down on the multi-layered network from above. Then, click and drag the network from the lower left to the upper right of the network view window to show the network in a layered view.

### Case Study II: Responses to oral glucose challenge across protein phosphorylation, transcriptome, and metabolome layers in the liver of wild-type and obese mice

Our second example is a trans-omic network in the wild type (WT) and obese (*ob*/*ob*) mouse liver responding to oral glucose challenge^11^. This network includes gene expression layers that are not present in the Case Study I network. transomics2cytoscape can easily expand the number of layers in its visualization. We show an automatic visualization of a trans-omic network that integrates protein phosphorylation, transcriptome and metabolome data obtained from the liver of WT and *ob*/*ob* mice.

**Fig. 5:**
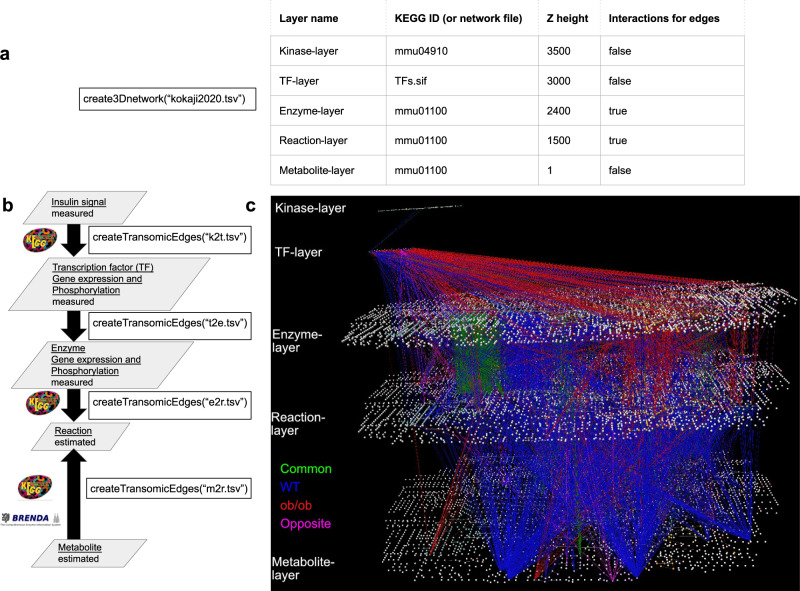
Transomics2cytoscape visualization of a five-layered trans-omic network of the responses to oral glucose challenge in the mouse liver. **a** A layer definition table file (‘kokaji2020.tsv’) that defines names, positions (‘Z height’), etc. of five layers. Note that the layers are functional classes of molecules and do not necessarily correspond to omic layers. The Z-coordinates for each layer were estimated and set based on the X-coordinate length of the KEGG global metabolic pathway map, as in Fig. 4a. In the network figure (**c**), the Z-coordinate for the enzyme layer was set to be 2,400, which is half of the X-coordinate length (4800) of the KEGG global metabolic map. The Z-coordinate distance between the enzyme layer and the top layer was narrower. We set the Z height for the top layer to be 3500. **b** The arguments for the function createTransomicEdges such as ‘k2t.tsv’, ‘t2e.tsv’, ‘e2r.tsv’, and ‘m2r.tsv’ are names for trans-omic interaction table files expected to include kinase-substrate (TF) relationships, TF-enzyme (target gene) relationships, enzyme reaction relationships, and metabolite-reaction (enzyme) interactions, respectively. **c** The trans-omic network of the responses to glucose challenge in the liver of WT and *ob*/*ob* mice^11^ automatically produced by transomics2cytoscape. The top layer is the insulin signaling pathway imported from KEGG (mmu04910). The second layer from the top is for transcription factors. The third to fifth layers from the top are the global metabolic pathways of mice from KEGG (mmu01100), representing enzyme genes, metabolic reactions, and metabolites, respectively. The edges between each layer are trans-omics interactions from signaling molecules to transcription factors (between layers 1 and 2), from TFs to target enzyme genes (between layers 2 and 3), from enzyme genes to metabolic reactions (between layers 3 and 4), and from metabolites to metabolic reactions (between layers 5 and 4). The edges in blue, red, green, and magenta indicate regulation functioning: only in WT, only in *ob*/*ob*, in similar ways both in WT and *ob*/*ob*, and in opposite ways in WT and *ob*/*ob*, respectively.

The network has trans-omics interactions between five layers, i.e., from signaling molecules to transcription factors (TFs), from TFs to enzymes, from enzymes to reactions, and from metabolites to metabolic reactions. The five layers of the network are the KEGG Insulin signaling pathway (mmu04910), a layer for TFs (tfs.sif file), and the KEGG global metabolic pathway (mmu01100) for the remaining three layers (enzymes, reactions, and metabolites). We created the trans-omic network of the glucose response in the mouse liver (Fig. 5) using datasets in the folder named usecase2 at 10.5281/zenodo.8201898 and codes in the folder named vignettes at 10.5281/zenodo.8201898.

### Testing for limitations in transomics2cytoscape analysis

To determine whether there are computational limitations in the transomics2cytoscape analysis, we duplicated the visualization of Case Study I and stacked from two to six networks along the Zaxis. We measured the time required for drawing the two to six networks (Supplementary Fig. 1). The time increased approximately linearly with the number of networks. This result implies that the rendering of the networks does not limit the realistic use of transomics2cytoscape because the six-fold network cannot be interpreted anymore in a standard monitor’s display space. We conducted this computational experiment on a Windows PC [12th Gen Intel Core i7-12700 2.10 GHz, 128 GB RAM, and Nvidia GeForce RTX 4070] with a monitor display resolution [WQHD(3440 × 1440)].

### Comparing transomics2cytoscape with existing tools

There exist several tools for 2.5D visualization; however, there has been no tool that automates the workflow for stacking network layers with conventional pathway layouts and visualizing the regulation between multiple layers. Using tools for 2.5D visualization, users can identify and understand novel pathways across multiple omic layers such as is shown in ref. ^4^. There are four requirements for software visualizing a trans-omic network:

1. Importing pathway map files provided by pathway databases [e.g., KGML files of KEGG]
2. Stacking 2D pathways as layers of a 2.5D network
3. Drawing edges between stacked layers
4. Automated workflow

Compatibility with pathway resources provided by KEGG is indispensable because the KEGG PATHWAY is one of the most commonly used and referred resources among various pathway databases. VANTED-HIVE^12^ was employed in the trans-omic network visualization by Yugi and colleagues^4^. The requirement ‘1’ shown above is provided by its original software VANTED, and ‘2’ and ‘3’ are realized in its extension VANTED-HIVE. Other software, such as Arena3D^13^ and BioLayout Express 3D^14^ were also considered for this purpose by the authors of ref. ^4^ but were not satisfactory in some aspects. Even after about 10 years and the emergence of multi-layered network visualization software (Arena3Dweb^15^ and its Cytoscape app^16^, Multipath^17^, and OmicsNet^18^) and powerful 3D network visualization software (Graphia^19^), there is still no software that satisfies all aspects of the four requirements (Table 1).

**Table 1:** Comparison of transomics2cytoscape with existing tools

	transomics2cytoscape	VANTED-HIVE	Arena3Dweb Cytoscape app	Multipath	OmicsNet	Graphia	BioLayout express 3D
Application type of 3D visualization	R package (Depends on RCy3 R package and Cy3D and KEGGscape Cytoscape apps)	An Add-on for the VANTED system	Cytoscape app (Depends on Arena3Dweb application)	R package (Depends on mully R package)	Web application	Desktop application	Desktop application
Limitations in trans-omics research project		No automation	No conventional layout in the network layer	No conventional layout in the network layer	No conventional layout in the network layer	No automation. No default stacked layers layout.	No automation. No default stacked layers layout.
Is actively maintained	+	–	+	+	+	+	–
Requirements for the visualization of a trans-omic network							
Importing pathway map files provided by pathway databases	+ KEGG KGML	+ KEGG KGML	+ Depends on the Cytoscape environment	+ BioPAX	+ KEGG, Recon3D, AGORA, EMBL GEMs	–	–
Stacking 2D pathways as layers of a 2.5D network	+	+	– Arranging one complex network into a multi-layered network instead of stacking pathways	– Arranging one complex network into a multi-layered network instead of stacking pathways	– Arranging one complex network into a multi-layered network instead of stacking pathways	–	–
Drawing edges between stacked layers	+	+	– Relocating some edges in a complex network instead of adding edges between stacked layers	– Relocating some edges in a complex network instead of adding edges between stacked layers	– Relocating some edges in a complex network instead of adding edges between stacked layers	– No example to make it automatic	– No example to make it automatic
Automated workflow	++	–	+ Not for trans-omics	+ Not for trans-omics	+ Not for trans-omics	–	–
3D network visualization							
Layer classification	KEGG pathway or user-defined network	KEGG pathway or user-defined network	Cytoscape node attribute	BioPAX class	Microbes, Metabolites, Genes/proteins, SNPs	–	–
Conventional network layout in layers	+ Automatic reproduction of KEGG layout in layers	+ Automatic reproduction of KEGG layout in layers	–	–	–	–	–
Directed networks (Edge target arrow shape)	– Cy3D does not support the visualization of the edge target arrow shape.	– We could not reproduce the environment needed for that verification.	+	+	–	+	– Target arrow shape is only supported in 2D visualization.
Automation of the data integration and visualization workflow							
Automation target	Trans-omics pathway stacking	–	Separating a STRING network into layers	Separating a BioPAX network into layers	Separating multi-omics data into layers	–	–
Automation customizability	Scriptable with Cytoscape Automation	–	Scriptable with Cytoscape Automation	Scriptable with mully R package	–	–	–
Data integration target	KEGG pathways and molecular interactions	Molecular interactions and biological images	Molecular interactions	Interaction between BioPAX entities and entries of DrugBank and UniProt	Protein-protein, miRNA-gene, metabolite-protein, and TF-gene interactions in some species	Molecular interactions	Molecular interactions
Input data of the automated workflow	List of network files supported by Cytoscape (or KEGG ID), List of Trans-omic interactions	–	Query to stringApp	BioPAX, DrugBank ID, UniProt ID	List of molecule IDs	–	–

A feature unique to transomics2cytoscape is that it allows automatic visualization of biochemical networks in a stacked-layer manner after importing conventional pathway layouts. Symbols used for feature evaluations with ‘−’ for absent, and ‘+’ for a more quantitative assessment (more ‘+’ indicates better support).

One limitation of VANTED-HIVE is that it does not support requirement ‘4’, which is the automation of the workflow for visualizing trans-omic networks. Visualization of the trans-omic network in ref. ^6^ demanded a considerable amount of manual effort and was dependent on the users’ personal skills and patience. In addition, VANTED-HIVE does not appear to be maintained anymore. Arena3Dweb is an interactive web tool that visualizes multi-layered networks in 3D space. The workflow of its Cytoscape app is designed to arrange one Cytoscape network, which is assumed to be obtained from STRING^20^ with stringApp^21^, into a multi-layered network visualized in Arena3Dweb. Each node of the Cytoscape network is assigned to one of the layers according to node attributes such as proteins and small compounds. However, Arena3Dweb Cytoscape app allows neither stacking pathways as we did in our Case Study I and II nor conserving conventional pathway layouts. Likewise, Multipath and OmicsNet cannot stack pathways or conserve conventional pathway layouts. Multipath is an R package to create a multi-layered network from BioPAX^22^. It depends on the mully^23^ R package to create, modify, and visualize multi-layered networks. OmicsNet is a web platform for multi-omics integration and 3D network visualization. It can perform workflows from data integration of multi-omics data to 2.5D multi-layer network visualization. Both Multipath and OmicsNet creates multi-layered networks by assigning network nodes into layers, but cannot stack pathways as layers. Furthermore, the layout of the network on each layer is limited to automatic coordination. The users cannot import conventional biochemical pathway layouts such as those in the KEGG PATHWAY database. Graphia and BioLayout Express3D provide generic 3D network visualization, but cannot visualize networks in layers. Also, they do not have any database integration or automation infrastructure that would help in realizing our four requirements.

### Prior knowledge is necessary for creating an interpretable network

**Fig. 6:**
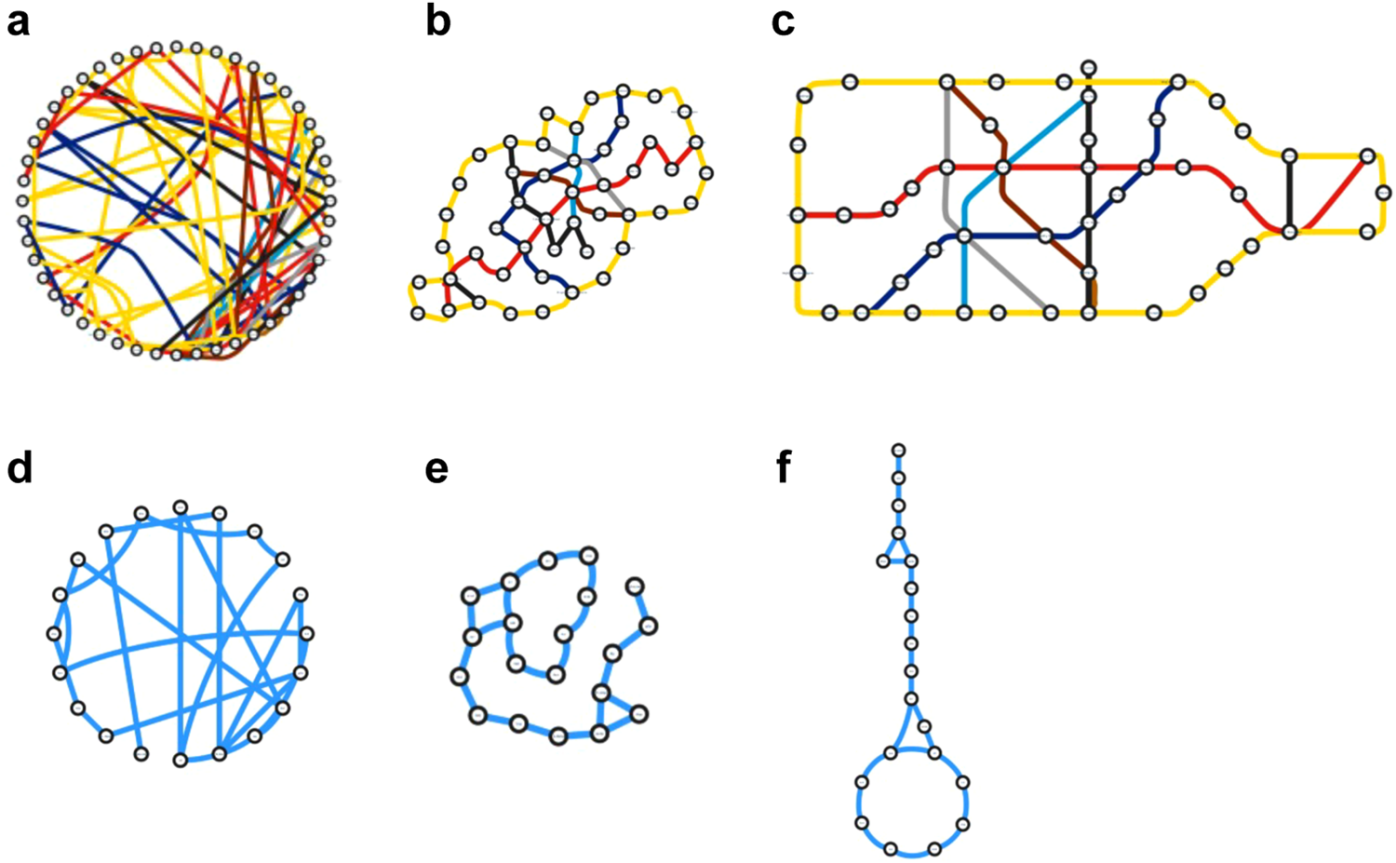
Unbiased network layouts are not necessarily more easily interpretable than conventional layouts. **a**–**c** The London underground network inside Circle Line before 2009 in three different layouts. The network layouts **a**, **b** are generated from the conventional layout **c** based on degree sorted circle layout and compound spring embedder (CoSE^16^), respectively. **d**–**f** The glycolysis and the tricarboxylic acid cycle in three layouts as in **a**–**c**. These images show that the auto-layout algorithms in common use produce layouts that differ significantly from the conventional pathway layouts.

People are likely to perceive, recognize, and interpret networks dependent on their prior knowledge, which is implemented as conventional network layouts. Do readers immediately find what the networks in Fig. 6a, b represent? These networks are the London underground network inside the Circle Line redrawn based on algorithms named degree sorted circle layout and compound spring embedder (CoSE^24^), respectively. Residents in London are expected to recognize their present location, destination, and the transfer stations in reference to the Circle Line in the original conventional layout (Fig. 6c). However, it is difficult to find where the Circle Line is in the algorithmically optimized network layouts.

Prior knowledge is similarly essential in recognition of biological networks. The networks in Fig. 6d, e depict central carbon metabolism redrawn by degree sorted circle layout and CoSE, respectively. In the conventional pathway layout (Fig. 6f), biologists working on metabolism might recognize the positions of the metabolites and the enzymes of interest in reference to the TCA cycle in the lower part of Fig. 6f, though it is difficult with the distorted TCA cycle in the algorithmically generated network layouts, which are not distinguishable from the underground network (Compare Fig. 6a, d).

If a network layout is shared as prior knowledge in a certain community, the network should be visualized according to the shared prior knowledge but not in a hairball layout or another algorithmically generated layout. KEGG pathway maps are used as such shared prior knowledge, particularly in the metabolism field. Import of the KEGG layouts to software facilitates the prior knowledge-based visualization and interpretation of networks.

## Discussion

The transomics2cytoscape package enables automatic visualization of 2.5D networks in conventional pathway layouts. Using the package, it is now possible to draw trans-omic networks at any step of a multi-omics/trans-omics research project, thereby allowing access to preliminary networks at each step. This information may provide hints to explore novel molecular mechanisms across multiple omic layers.

We specified the four requirements for the human-interpretable and reproducible visualization of multi-layered biochemical networks: (1) importing pathway maps from databases, (2) stacking pathways as layers, (3) drawing edges between stacked layers, and (4) automation of workflow. Each of them has already been realized by other biochemical network visualization software but not sufficient for the human-interpretable and reproducible visualization of multi-layered biochemical networks. The transomics2cytoscape package showed that simultaneously satisfying the four requirements allows the human-interpretable and reproducible visualization of multi-layered biochemical networks.

The concept behind the workflow of transomics2cytoscape is to conserve the conventional layout of nodes and edges in the pathway layers. This is different from the workflows of other automated software. The transomics2cytoscape package adds edges between layers to understand molecular interactions between pathway layers defined by prior knowledge. On the other hand, the other tools do not conserve conventional pathway layouts (Table 1). Instead, they rearrange nodes of a single-layered complex network into multiple layers to find associations between certain groups of molecular species. Consequently, their layers are often occupied by mutually isolated nodes but not by a pathway.

The transomics2cytoscape package automates only the 2.5D visualization part of trans-omics analysis, but not other processes such as omic data integration. Automation of the workflow of trans-omics analysis before and after visualization, which is described below, will be presented in our future works. We plan to provide a workflow execution environment that summarizes all of these processes.

The connectivity data of trans-omic networks are provided after reconstruction procedures, but these are not fully automated yet. We have not yet automated the estimation of Z-coordinates in Case Study I and II. Fully automated network reconstruction and connecting with transomics2cytoscape will provide a seamless end-to-end pipeline from omic data to visualization. We have established a workflow of network reconstruction that includes identifying changed molecules from multiple omic data and connecting the changed molecules based on prior knowledge, such as publicly available pathway databases. Automation of the procedure has already been prototyped in our group using the Garuda Platform^25^, which allows cooperative execution of multiple software packages that constitute each step of the end-to-end pipeline. We plan to define file formats that facilitate data exchange and cooperative procedures with the Garuda-compatible pathway reconstruction software.

A reconstructed 2.5D network is also an information-rich representation from which to extract biological relevance. Kokaji et al.^11^ developed a technology to summarize 2.5D networks by using enrichment analysis for congregating a group of molecules into a name of a pathway. For example, in a case where the fraction of dynamically expressed genes in the glycolysis pathway is significantly larger than in other pathways, and the genes encoding the enzymes are regulated by a common transcription factor X, it can be summarized “enzyme expression in glycolysis is significantly changed by regulation under X”. We will consider implementing this summarizing function in the future.

Currently, transomics2cytoscape supports only the KEGG ID and pathway file format. In the future, we will extend this to other pathway databases such as Reactome^26^ and Wikipathways^27^. An alternative format is Systems Biology Graphical Notation (SBGN), which provides a universal notation rule for intracellular biochemical processes such as metabolism, gene expression regulation, signal transduction, and other regulatory pathways^28^. Large-scale pathway maps such as EGF signaling by Oda et al.^29^ and the Parkinson’s disease-related pathway by Fujita et al.^30^ have already been published in the SBGN format. For 2D pathway networks, there are studies that realize interoperability between multiple visualization tools through standard formats such as SBGN. On the other hand, visualization tools for 3D networks such as Arena3Dweb Cytoscape app, Multipath, OmicsNet, and transomics2cytoscape do not provide interoperability with each other. This is because there is no common data format for multi-layered networks. We recognize the need to develop software for data conversion to facilitate interoperability between these tools in the future. Interactions between omic layers can also be obtained from databases such as BRENDA as well as pathway data from KEGG. However, transomics2cytoscape has not yet automated the retrieval processes for inter-layer edges. This would be realized by creating trans-omics interaction table files shown in Fig. 2b from databases such as OmniPath^31^, SIGNOR^32^, and IntAct^33^.

We are considering future addition of functions that support generating 3D movies of trans-omic networks, such as previously presented in the study of insulin action in rat hepatoma Fao cells^4^, in which one of the present authors participated. The movie first shows the perspective of the trans-omic network and then shows close-ups of each pathway and molecule so that viewers can be aware of the connection between the entire network and each component. The movie was authored using a 3DCG software Shade (https://shade3d.jp). However, the authoring process was labor-intensive and required expertize specific to the software. Further automation will be needed to make the movie-authoring technology accessible to a broader usership. CyAnimator, a plug-in of Cytoscape, can automate pathway video creation^34^. However, it only supports pathways on a 2D plane and the development community is not currently very active. The BioLayout Express 3D mentioned above also has the ability to create animations. Example movies that were made six years ago are available on YouTube (https://www.youtube.com/channel/UCLUnA39yq1C0pmRNU40NRHQ). Another possibility in this area is future extension of Cy3D^35^, which is used inside transomics2cytoscape.

Although it would be another work entirely after implementing 2.5D network movie generator, it will be the next challenge for large-scale pathway visualization to provide virtual reality (VR) experiences in the trans-omics network for knowledge discovery. The VRNetzer platform is a software that enables users to experience large-scale molecular networks via VR headsets^36^. Cy2VR (https://github.com/larsjuhljensen/Cy2VR) is a tool to convert a Cytoscape network into a VR experience. In addition, a paper on visualization methods for interpreting genome-wide association study data in VR has already been published^37^. VR for visualization of single-cell data has also been presented^38^. It is worth keeping an eye on the trend of developments regarding what humans can find by wearing headsets and moving around in a large-scale network and related omics data.

In conclusion, our transomics2cytoscape package satisfies all of the four requirements for the human-interpretable and reproducible visualization of multi-layered biochemical networks: (1) importing pathway maps from databases, (2) stacking pathways as layers, (3) drawing edges between stacked layers, and (4) automation of workflow. By these features, users are allowed to frequently visualize 2.5D networks in conventional pathway layouts during their multi-omics/trans-omics projects so that one can identify and understand novel pathways across multiple omic layers.

## Methods

### Software components used in transomics2cytoscape

Transomics2cytoscape uses KEGGREST to retrieve KGML (KEGG pathway XML). The transomics2cytoscape package in R uses RCy3^39^ to automate^40^ importing multiple pathway maps and integrating them into a 2.5D network in Cytoscape. Also, the Cytoscape Apps KEGGscape^41^ and Cy3D are used for KEGG pathway import and 2.5D network visualization, respectively.

### Dataset preprocessing

The connectivity data of the trans-omic networks shown in Yugi et al.^4^ and Kokaji et al.^11^ were obtained from supplementary datasets of the original articles, in particular Table S3 (https://www.cell.com/cms/10.1016/j.celrep.2014.07.021/attachment/5b83010d-f4f0-4c88-829f-6ceea27fd1e8/mmc4.xlsx) and Table S4 (https://www.cell.com/cms/10.1016/j.celrep.2014.07.021/attachment/833df0b2-8abc-4229-aa97-96d07f8579ba/mmc5.xlsx) of Yugi et al. and Data file S11 (https://www.science.org/doi/suppl/10.1126/scisignal.aaz1236/suppl_file/aaz1236_data_file_s11.xlsx) of Kokaji et al. The IDs of molecules were converted into KEGG–compatible IDs such as KEGG gene ID and EC numbers by using Ensembl BioMart^42^ and ‘Database to Database Conversions’ provided by bioDBnet^43^ in case cross reference tables were not available. The data used in this study are available in our GitHub repository https://github.com/ecell/transomics2cytoscape.

### Reporting summary

Further information on research design is available in the Nature Research Reporting Summary linked to this article.

### Supplementary information

Supplementary Figure 1

Reporting summary

## Data Availability

Transomics2cytoscape and the data used in this study can be found on GitHub at https://github.com/ecell/transomics2cytoscape under the Artistic-2.0 license. The datasets and codes (vignettes) used in Case Studies I and II are also available at Zenodo (10.5281/zenodo.8201898).

## Code availability

Transomics2cytoscape is open source and can be found on GitHub at https://github.com/ecell/transomics2cytoscape under the Artistic-2.0 license.
