# Supplementary material for: Transomics2cytoscape: an automated software for interpretable 2.5-dimensional visualization of trans-omic networks
Source: NPJ Syst Biol Appl. 2024 Feb 19;10:16. doi: 10.1038/s41540-024-00342-8 (PMC10876688; doi:10.1038/s41540-024-00342-8)
Supplement: Supplementary file 1 — Supplementary Figure 1 [file 41540_2024_342_MOESM1_ESM.pdf]

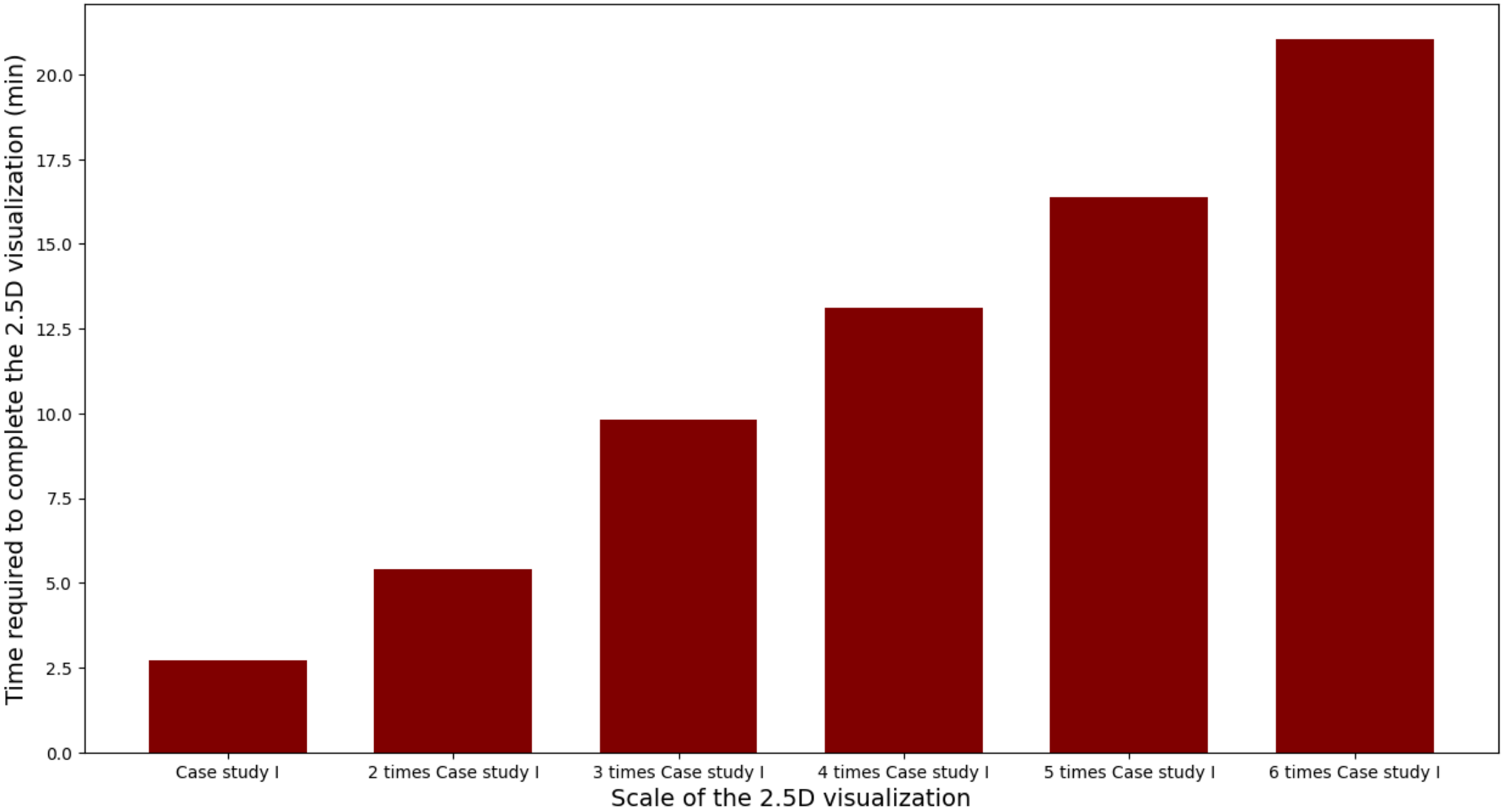

Supplementary Figure 1. The time required to complete the visualization as the network scale increases. The X-axis represents the network scale in terms of how many networks of Case Study I are visualized at once. The Y-axis represents the time needed for the visualization of the networks.
